# Efficacy and safety of antivirals in treating hearing loss: A systematic review and network meta-analysis

**DOI:** 10.3389/fneur.2022.1027615

**Published:** 2022-12-16

**Authors:** Li-Mei Liu, Li-Li Xia

**Affiliations:** Pharmacy Department of Chongqing YouYou BaoBei Women's and Children's Hospital, Chongqing, China

**Keywords:** antivirals, hearing loss, systematic review, network meta-analysis, effectiveness

## Abstract

**Objectives:**

This study aimed to compare and rank the therapeutic effects of antivirals in treating hearing loss using a network meta-analysis approach.

**Methods:**

We searched the PubMed, Embase, and Cochrane Library databases to identify eligible randomized controlled trials (RCTs) through April 2022. Placebo-controlled or head-to-head RCTs of three categories of antivirals for hearing loss were included, and pooled relative risks (RRs) with 95% confidence interval (CI) were calculated using pairwise and network meta-analyses.

**Results:**

Six RCTs with 405 patients were included in the final analysis. The results showed that ganciclovir had relatively better effects on the incidence of hearing recovery (surface under the cumulative ranking: 88.8%) compared with other antivirals. However, pairwise comparison analyses found that the use of antivirals significantly increased the incidence of hearing recovery compared with the use of a placebo (RR: 1.27; 95% CI: 1.04–1.54; *P* = 0.017), while no significant difference was observed between any two categories of antivirals. Finally, the use of antivirals did not increase the risk of adverse events compared with the use of a placebo (RR: 1.27; 95% CI: 0.82–1.98; *P* = 0.285).

**Conclusion:**

Antivirals are more efficacious than placebos for hearing recovery in patients with hearing loss, and ganciclovir is the most likely to increase the incidence of hearing recovery.

## Introduction

Hearing loss is characterized by the deterioration of hearing acuity and is associated with an elevated hearing threshold. Studies have demonstrated that hearing loss is significantly related to physical (1), cognitive (2), and psychosocial outcomes (3) and accounts for higher medical expenses (4). The World Health Organization indicates that more than 5% of individuals are affected by hearing loss, especially patients older than 65 years (5). The common risk factors for hearing loss include viral infections, microcirculatory disorders, autoimmune disorders, and labyrinthine hemorrhage (6–9). The disease spectrum of hearing loss, including sensorineural hearing loss (SNHL) and noise-induced hearing loss, could be preventable and treatable (9–11).

Nowadays, the use of antivirals has not been recommended for improving hearing loss in patients with isolated SNHL or asymptomatic congenital cytomegalovirus (CMV) disease (12, 13). A systematic review performed by De Cuyper et al. (14) identified 18 studies that revealed that the use of (val) ganciclovir could improve hearing loss and deterioration in children with symptomatic congenital CMV disease. A Cochrane review identified four randomized controlled trials (RCTs) with a low risk of bias and found that the use of antivirals had no significant effect on hearing improvement in idiopathic sudden SNHL (15). These studies focused on qualitative analyses, and the therapeutic effects of antivirals were neither compared nor ranked on the basis of direct and indirect evidence. Therefore, the current systematic review and network meta-analysis were conducted to update and expand previous systematic reviews to inform clinical practice by comparing different types of antivirals for managing hearing loss.

## Materials and methods

### Search strategy and selection criteria

This systematic review and network meta-analysis were performed according to the Preferred Reporting Items for Systematic Reviews and Meta-Analysis guidelines (16). Placebo-controlled or head-to-head RCTs of the three categories of antivirals for treating hearing loss were included in the analysis. The PubMed, Embase, and Cochrane Library databases were searched for trials that met the inclusion criteria through April 2022, and the publication language and status were not restricted. The following search terms were used as text words or medical subject heading terms: (“antiviral agent” OR “acyclovir” OR “valacyclovir” OR “ganciclovir”) AND (“cytomegalovirus infection” OR “hearing loss”). Additional trials that were completed but not published were searched on the ClinicalTrials.gov website (NIH, USA). We also manually reviewed reference lists of relevant reviews and articles to identify additional eligible trials.

The literature search and study selection were independently conducted by two authors, and conflicts between the authors were resolved through mutual discussion. Studies were included if they met the following criteria: (1) patients: all of patients diagnosed sudden sensorineural hearing loss or congenital CMV disease and were not restricted by age (17); (2) intervention: acyclovir, valacyclovir, or ganciclovir; (3) control: placebo or no treatment; (4) outcome: audiologic outcomes and adverse events; and (5) study design: had to have an RCT design.

### Data collection and quality assessment

The following items from each trial were abstracted by two authors: first author's name, publication year, region, sample size, mean age, proportion of males, inclusion criteria, intervention, control, treatment duration, and reported outcomes. Subsequently, these two authors assessed the methodological quality of the included trials using a risk of bias approach according to the methods described by the Cochrane Collaboration, which includes seven specified domains: random sequence generation, allocation concealment, blinding of participants and personnel, blinding of outcome assessment, incomplete outcome data, and other (18). Inconsistent results regarding data extraction and quality assessment between authors were settled by an additional author referring to the original article.

### Statistical analysis

Therapeutic effects of antivirals were assigned as continuous and dichotomous data, and pooled RRs with 95% CIs were calculated as effect estimates. Indirect and mixed comparisons in network meta-analysis were analyzed to compare different antivirals (19), and the loop-specific approach was applied to assess differences between direct and indirect estimates for a specific comparison in the loop (20). The design-by-treatment interaction inconsistency model was used to assess the assumption of consistency in the entire network (19). In this study, an inconsistent model was applied because of the underlying heterogeneity across the included trials. To rank various antivirals for the investigated outcome, the surface under the cumulative ranking (SUCRA) probabilities were used (21). Publication bias was assessed using comparison-adjusted funnel plots (22). Subsequently, pairwise comparison analyses were performed using the random-effects model (23, 24). *I*^2^ and Q statistics were used to assess heterogeneity across the included trials, and significant heterogeneity was defined as *I*^2^ > 50.0% or *P* < 0.10 (25). The inspection level for the pooled results was two sided, and *P* values < 0.05 were considered statistically significant. Statistical analyses were performed using Software STATA (version 10.0; Stata Corporation, College Station, TX, USA).

## Results

### Literature search and study selection

**Figure 1:**
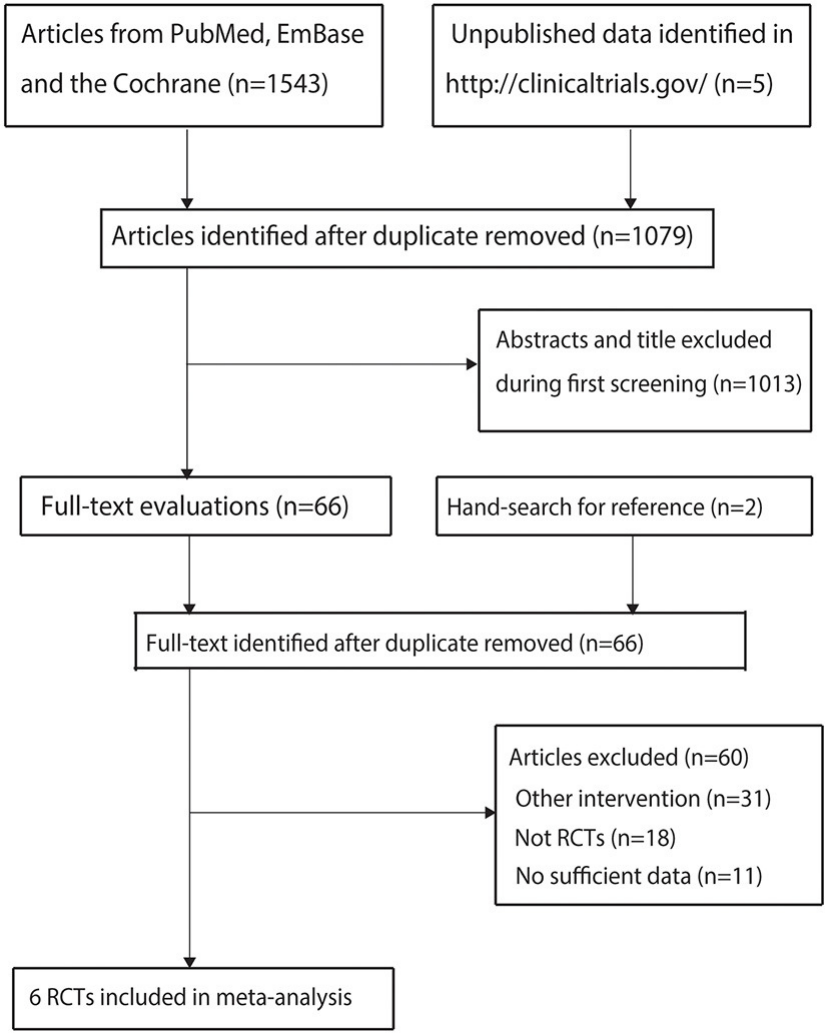
PRISMA flowchart with details of the literature search and study selection.

A total of 1,543 articles were identified from the initial electronic search, and 1,079 articles were retained after duplicate topics were removed. Subsequently, 1,013 articles were excluded because they investigated irrelevant topics. The remaining 66 studies were retrieved for further evaluation, and 60 studies were excluded because of other interventions (*n* = 31), not being RCTs (*n* = 18), and having insufficient data (*n* = 11). Reviewing the reference lists of relevant studies yielded two potentially eligible trials, and these two trials were obtained through electronic searches. Finally, six RCTs were selected for the final quantitative analysis (Figure 1) (26–31).

### Study characteristics

The baseline characteristics of the included trials and recruited patients are presented in Table 1. In the six included trials, the sample size ranged from 42 to 96, and treatment duration ranged from 7.0 days to 6.0 weeks. Four trials included adult patients, whereas the remaining two trials included children. Three trials investigated the therapeutic effects of acyclovir, two trials assessed the therapeutic effects of valacyclovir, and the remaining trial evaluated the therapeutic effects of ganciclovir. Table 2 presents the methodological quality of the included trials; four of the included trials had a low risk of bias, while the remaining two trials had a relatively high risk of bias.

**Table 1:** The baseline characteristics of included trials.

**Study**	**Region**	**Sample size**	**Age**	**Male (%)**	**Inclusion criteria**	**Intervention**	**Control**	**Treatment duration**	**Reported outcomes**
Stolroos et al. (26)	Netherlands	43 (22/21)	45.5 years	NA	ISSHL: cochlear hearing loss of unknown etiology; hearing loss of at least 30 dB HL for three subsequent one octave steps in frequency; hearing loss occurring within 24 h; blank otological history	Acyclovir (10 mg/kg, 3 times/day for 7 days)	Placebo	7.0 days	Hearing recovery, adverse events
Tucci et al. (27)	USA	94 (50/44)	55.8 years	53.6	SSHL: loss of at least 30 dB in 3 contiguous frequencies over a period of < 3 days in patients who have been monitored previously for hearing loss; subjective marked loss of hearing in patients with subjectively normal baseline hearing and no previous record of audiometry; patients seen within 10 days of onset of hearing loss; no underlying disease that could be associated with sudden sensorineural hearing loss as an etiologic factor	Valacyclovir (1 g, 3 times/day for 10 days)	Placebo	10.0 days	Hearing recovery, adverse events
Kimberlin et al. (28)	USA and Canada	42 (25/17)	9.2 days	54.8	Symptomatic congenital CMV disease involving the central nervous system, and CMV was confirmed from a urine specimen	Ganciclovir (6 mg/kg intravenously per 12 h for 6 weeks)	No treatment	6.0 weeks	Hearing recovery, adverse events, and death
Westerlaken et al. (29)	Netherlands	70 (37/33)	45.3 years	65.7	ISSHL: SSHL of unknown cause; hearing loss of at least 30 dB hearing level for 3 subsequent 1-octave steps in frequency in the standard pure tone audiogram; bland otologic history; and hearing loss occurring within a period of 24 h	Acyclovir (10 mg/kg, 3 times/day for 7 days)	Placebo	7.0 days	Hearing recovery
Uri et al. (30)	Israel	60 (29/31)	45.8 years	55.0	ISSHL: hearing loss of at least 20 dB in 3 contiguous frequencies within 7 days	Acyclovir (15 mg/kg per day for 7 days)	No treatment	7.0 days	Hearing recovery
Kimberlin et al. (31)	UK	96 (47/49)	< 30.0 days	NA	Symptomatic congenital CMV disease, irrespective central nervous system involvement, and CMV was detected in urine or throat-swab specimens by means of culture, shell-vial culture, or polymerase-chain-reaction assay	Valacyclovir (16 mg/kg, 2 times/day for 6 weeks)	Placebo	6.0 weeks	Hearing recovery, adverse events

CMV, cytomegalovirus; ISSHL, idiopathic sudden sensorineural hearing loss; NA, not available; SSHL, sudden sensorineural hearing loss.

**Table 2:** Methodological quality assessment of included trials.

**Study**	**Random sequence generation**	**Allocation concealment**	**Blinding of participants and personnel**	**Blinding of outcome assessment**	**Incomplete outcome data**	**Selective reporting**	**Other**
Stolroos et al. (26)	Unclear	Low	Low	Low	Low	Unclear	Low
Tucci et al. (27)	Unclear	Low	Low	Low	Low	Unclear	Low
Kimberlin et al. (28)	Unclear	Unclear	High	High	Low	Unclear	Low
Westerlaken et al. (29)	Unclear	Low	Low	Low	Low	Unclear	Low
Uri et al. (30)	Unclear	Unclear	High	High	High	Unclear	Low
Kimberlin et al. (31)	Low	Low	Low	Low	Low	Unclear	Low

### Network meta-analysis

**Figure 2:**
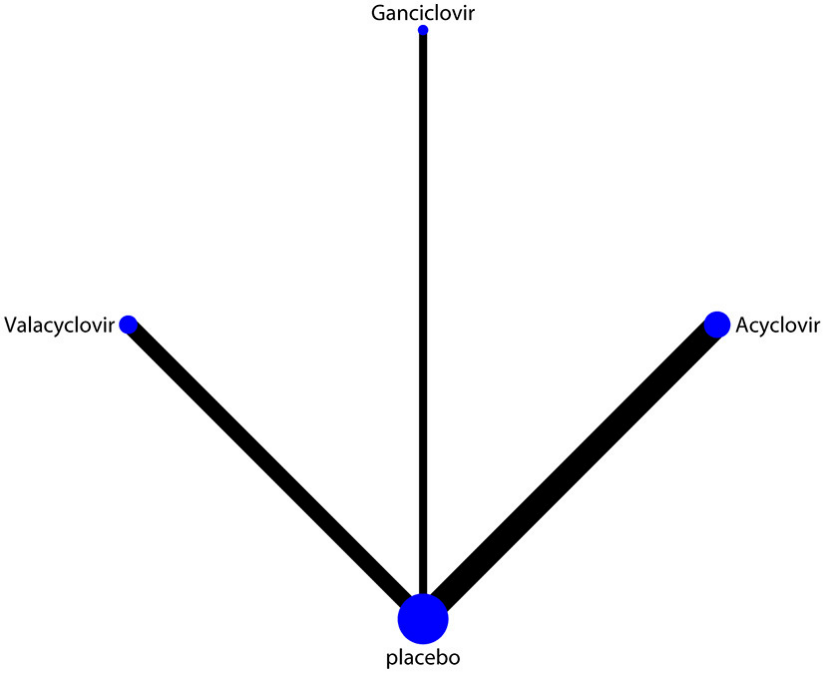
Network of comparisons for hearing recovery included in the analysis. The nodes were weighted by the number of trials, and the edges were weighted by the precision of the direct estimate for each pairwise comparison.

**Figure 3:**
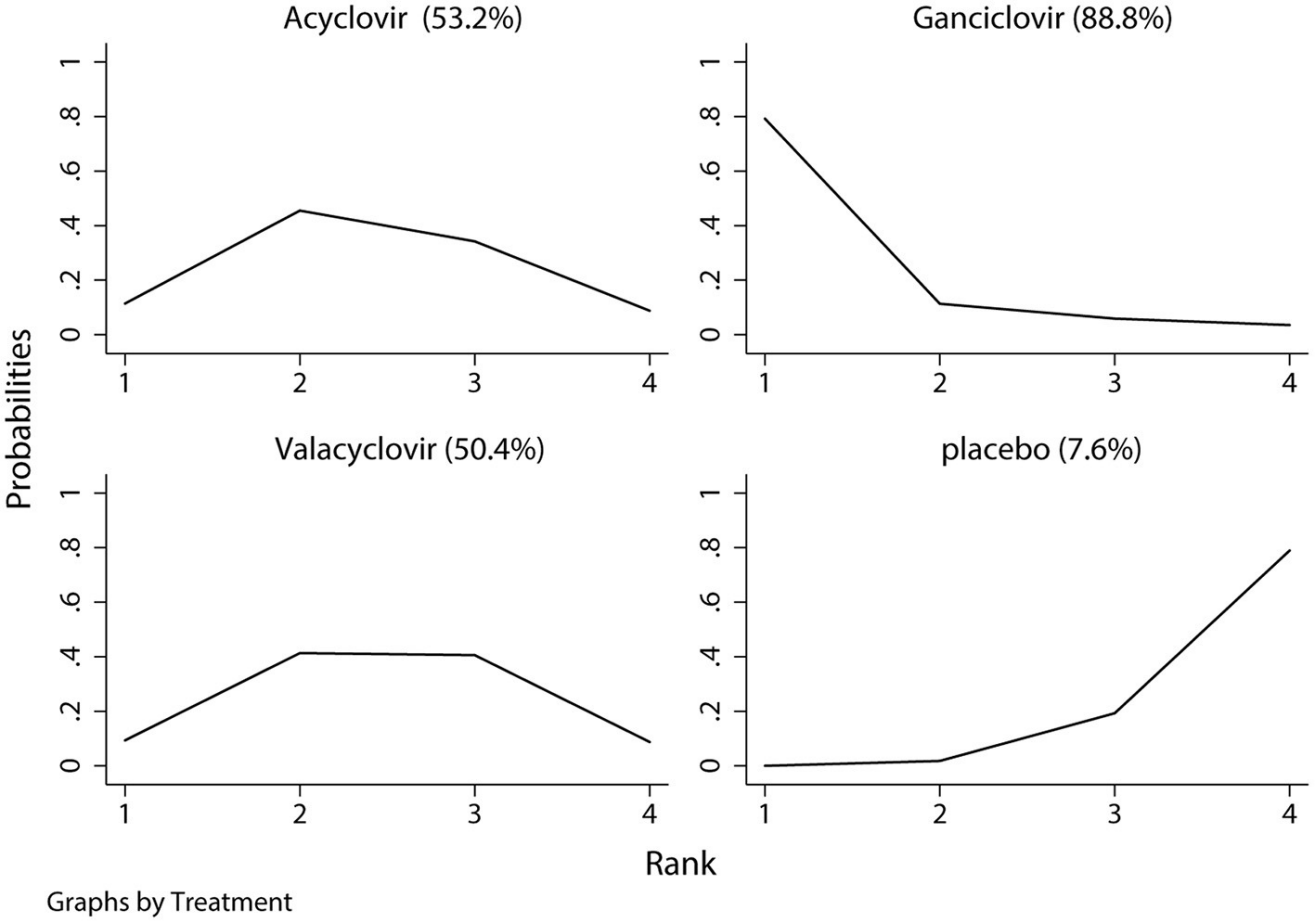
The surface under the cumulative ranking test for hearing recovery. Large area indicated better treatment effect on hearing recovery.

**Figure 4:**
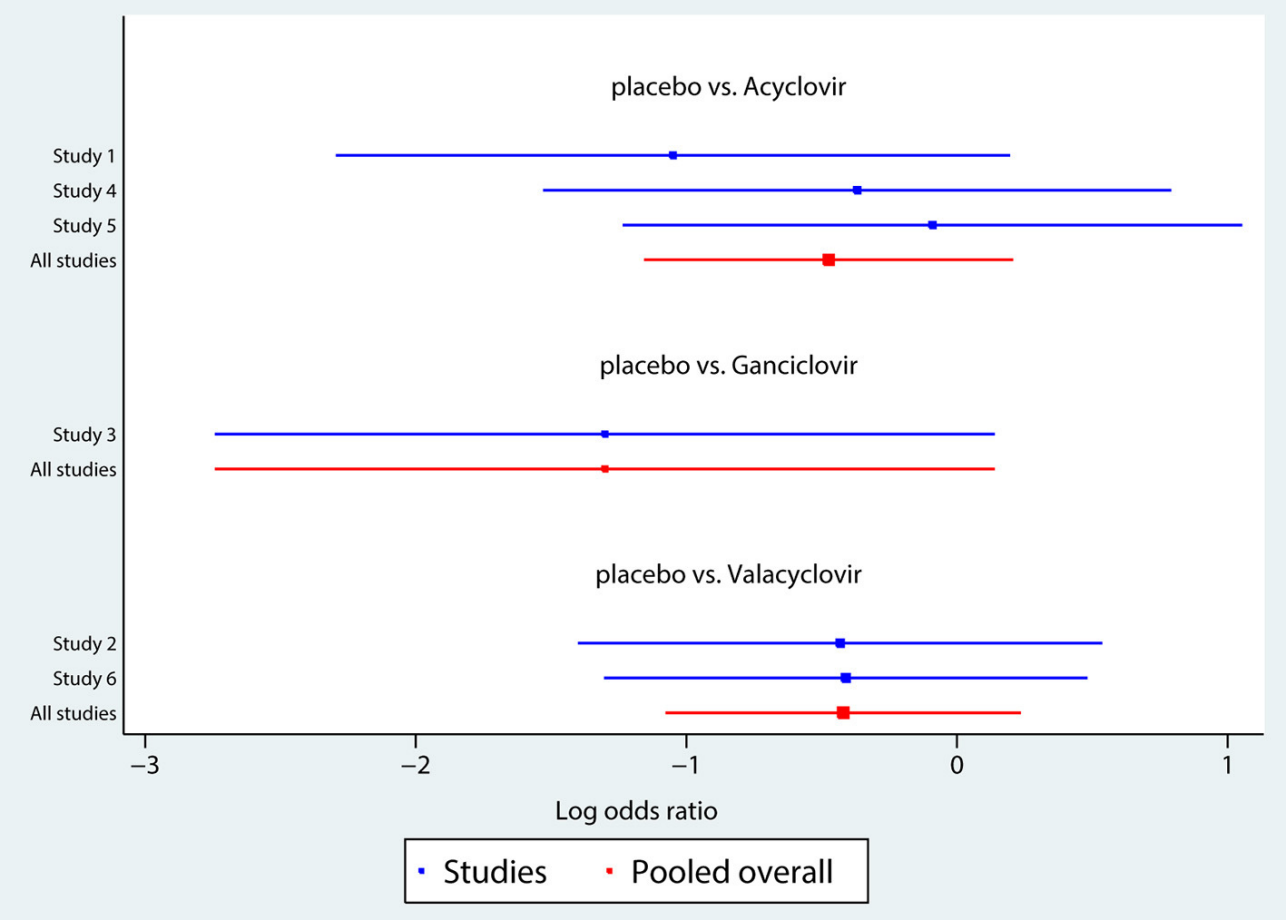
Pairwise comparison agents for hearing recovery.

**Figure 5:**
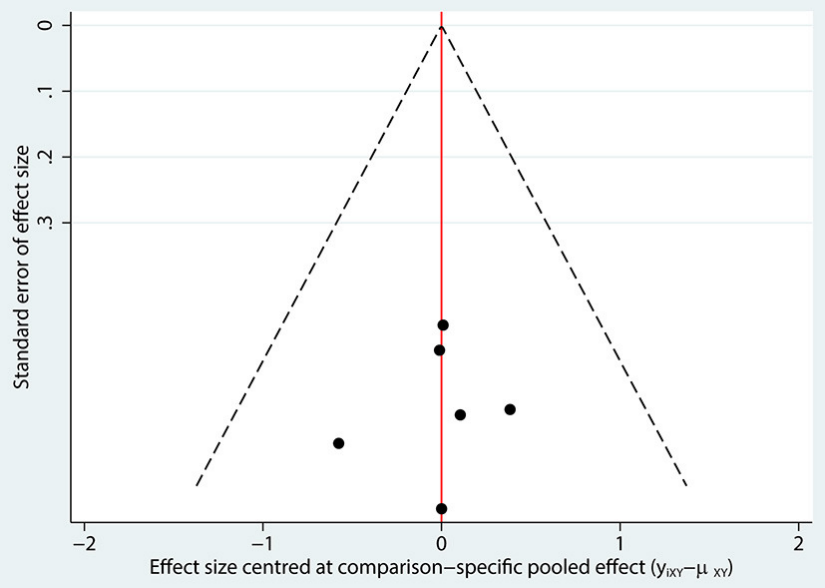
Funnel plot for hearing recovery.

The network of eligible comparisons of hearing recovery is shown in Figure 2. The nodes were weighted based on the number of trials assessed for each treatment and the edges were weighted based on the precision of the direct estimate for each pairwise comparison. The treatment effects of the antivirals were ranked using SUCRA probabilities. We noted that ganciclovir had relatively better treatment effects on the incidence of hearing recovery (88.8%; Figure 3). The results of pairwise comparisons of the study agents are provided in Figure 4 and Table 3. There were no significant differences among the three antivirals in terms of the incidence of hearing recovery. The review of the funnel plot revealed no evidence of publication bias for the effect of antivirals on the incidence of hearing recovery (Figure 5).

**Table 3:** Indirect comparison the therapeutic effects of antivirals on the incidence of hearing recovery.

**Intervention**	**RR (95%CI)**	***P*-value**	***I*^2^ (%)**	***P*-value for heterogeneity**
Acyclovir	1.39 (0.91–2.11)	0.125	0.0	0.734
Valacyclovir	1.17 (0.91–1.52)	0.221	0.0	0.838
Ganciclovir	1.43 (0.93–2.20)	0.107	–	–
Acyclovir vs. valacyclovir	1.19 (0.73–1.94)	0.493	–	–
Acyclovir vs. ganciclovir	0.97 (0.53–1.77)	0.926	–	–
Valacyclovir vs. ganciclovir	0.82 (0.50–1.35)	0.433	–	–

### Traditional meta-analysis

**Figure 6:**
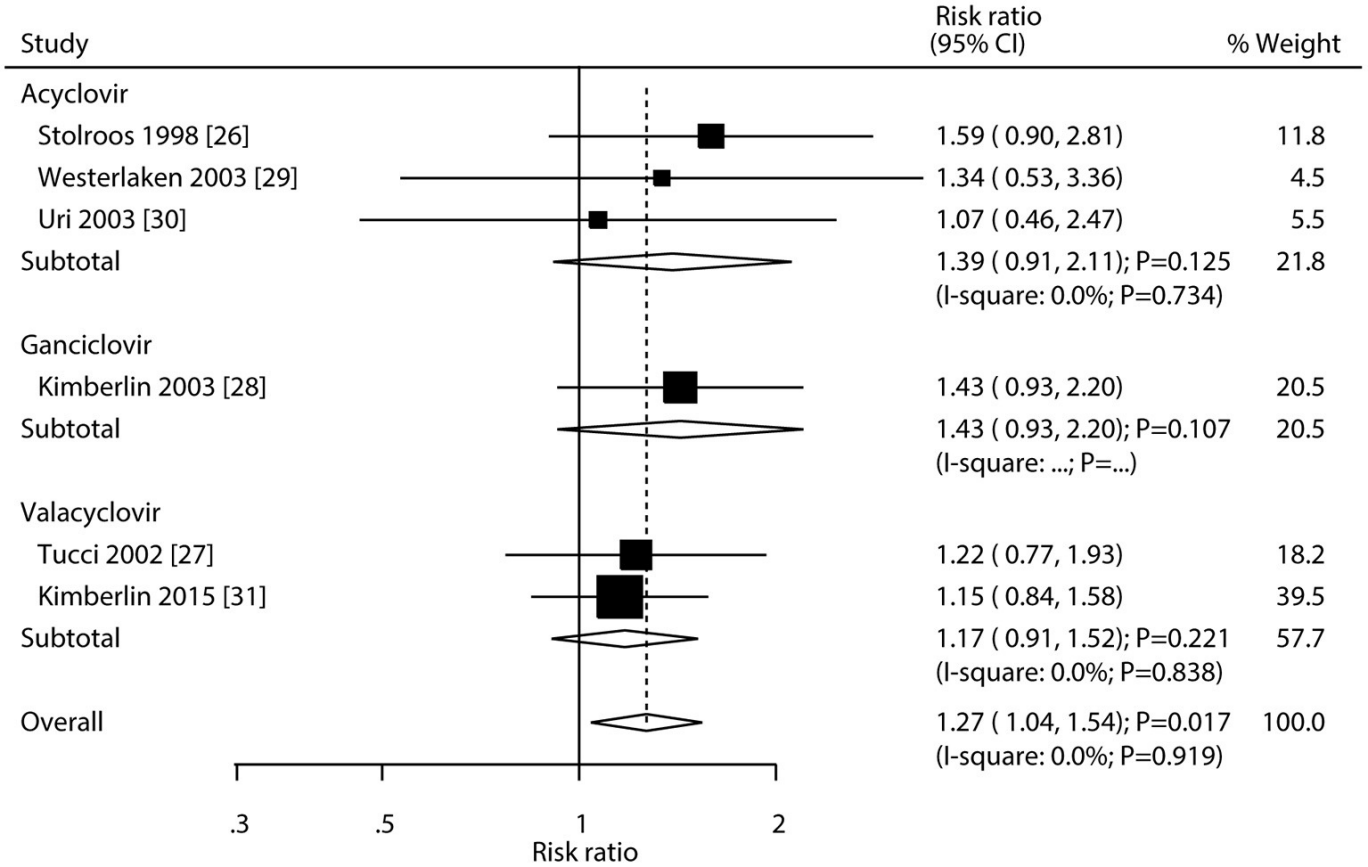
Forest plot of the meta-analysis of the incidence of hearing recovery between antivirals and placebo.

After pooling all included trials, we noted that the use of antivirals significantly increased the incidence of hearing recovery compared with the use of a placebo (RR: 1.27; 95% CI: 1.04–1.54; *P* = 0.017; Figure 6), and no evidence of heterogeneity was observed (*I*^2^ = 0.0%; *P* = 0.919). Moreover, subgroup analyses found that acyclovir (RR: 1.39; 95% CI: 0.91–2.11; *P* = 0.125), valacyclovir (RR: 1.17; 95% CI: 0.91–1.52; *P* = 0.221), and ganciclovir (RR: 1.43; 95% CI: 0.93–2.20; *P* = 0.107) were not associated with the incidence of hearing recovery compared with the placebo.

The summary results for adverse events are presented in Table 4. There were no significant differences in the risk of total adverse events between the antivirals and placebo (RR: 1.27; 95% CI: 0.82–1.98; *P* = 0.285). Moreover, the use of antivirals did not affect the risk of headache (RR: 2.86; 95% CI: 0.32–25.40; *P* = 0.345), nausea (RR: 0.95; 95% CI: 0.06–14.30; *P* = 0.973), stomach pain (RR: 0.32; 95% CI: 0.01–7.42; *P* = 0.476), elevated blood glucose (RR: 0.32; 95% CI: 0.01–7.42; *P* = 0.476), increased creatinine (RR: 2.87; 95% CI: 0.12–68.47; *P* = 0.515), increased total bilirubin (RR: 1.43; 95% CI: 0.61–3.31; *P* = 0.410), thrombopenia (RR: 1.37; 95% CI: 0.24–7.77; *P* = 0.725), neutropenia (RR: 1.57; 95% CI: 0.43–5.76; *P* = 0.493), and death (RR: 0.37; 95% CI: 0.10–1.37; *P* = 0.136).

**Table 4:** The summary results for adverse events.

**Outcome**	**RR (95%CI)**	***P*-value**	***I^2^* (%)**	***P*-value for heterogeneity**
Total adverse events	1.27 (0.82–1.98)	0.285	20.9	0.282
Headache	2.86 (0.32–25.40)	0.345	–	–
Nausea	0.95 (0.06–14.30)	0.973	–	–
Stomach pain	0.32 (0.01–7.42)	0.476	–	–
Elevated blood glucose	0.32 (0.01–7.42)	0.476	–	–
Increased creatinine	2.87 (0.12–68.47)	0.515	–	–
Increased total bilirubin	1.43 (0.61–3.31)	0.410	–	–
Thrombopenia	1.37 (0.24–7.77)	0.725	–	–
Neutropenia	1.57 (0.43–5.76)	0.493	86.5	0.006
Death	0.37 (0.10–1.37)	0.136	–	–

## Discussion

To our knowledge, no previous systematic review and network meta-analysis has compared and ranked the efficacy and safety of antivirals in the treatment of hearing loss. This comprehensive, quantitative, systematic review and network meta-analysis was based on six RCTs involving 405 patients presenting with hearing loss who were randomly assigned to treatment with antivirals (acyclovir, valacyclovir, and ganciclovir) or placebos. Hearing recovery and any adverse events following treatment were investigated. The study results show that ganciclovir had relatively better treatment effects on hearing recovery. However, pairwise comparison analyses indicated that the incidence of hearing recovery was not statistically significant when comparing any two types of antivirals. A traditional meta-analysis found that the use of antivirals significantly increased the incidence of hearing recovery compared with the use of a placebo. Finally, the use of antivirals did not increase the risk of adverse events compared with the use of a placebo in patients with hearing loss.

Three studies reported the therapeutic effect of acyclovir on the incidence of hearing recovery (26, 29, 30). All three trials included adult patients with idiopathic sudden SNHL and reported an increased incidence of hearing recovery when using acyclovir. Moreover, the use of valacyclovir did not yield a benefit on hearing in adult patients with idiopathic sudden SNHL (27). The potential reason for the treatment effects of antiviral drugs in adults can be explained by the main cause of idiopathic sudden SNHL, including circulatory disturbance, membrane rupture, and viral loads, and might be an important factor for the role of antiviral drugs in the incidence of hearing recovery (29). Moreover, vestibular involvement, severity of initial hearing loss, audiogram shape, dose of antivirals, and interval between the onset of hearing loss and beginning of treatment could affect the prognosis of idiopathic sudden SNHL (32, 33).

Two of the included trials reported the treatment effects of antiviral drugs on hearing recovery in newborns (28, 31). Kimberlin et al. (28) found that ganciclovir therapy during the neonatal period could prevent hearing deterioration. Moreover, Kimberlin et al. (31) indicated that valacyclovir could improve long-term hearing and developmental outcomes in newborns. This network-analysis indicated that ganciclovir was associated with relatively better treatment effects on hearing recovery, while pairwise comparison analyses did not find significant differences among the three antiviral drugs. This result could be explained by only one trial addressing the treatment effect of ganciclovir on the incidence of hearing recovery, and the incidence of hearing recovery was lower with a smaller number of included trials, which caused the power to be insufficient to detect potentially significant differences.

A traditional meta-analysis found that the use of antivirals significantly increased the incidence of hearing recovery compared with the use of a placebo. Moreover, although the incidence of hearing recovery in patients treated with ganciclovir was superior to that in patients treated with acyclovir and valacyclovir, the differences were not statistically significant. However, the result should be cautiously recommended because of a relatively high risk of bias in the methodological quality of the trial performed by Kimberlin et al. (28). Finally, we noted that antivirals were associated with a higher risk of headache, increased creatinine and total bilirubin levels, thrombocytopenia, and neutropenia, while the risks of nausea, stomach pain, elevated blood glucose levels, and death were lower in patients treated with antivirals. However, the difference in the risk of any specific adverse events between antivirals and placebo was not statistically significant. These results could be explained as follows: (1) steroids were used as a background therapy, and most adverse events were related to the use of steroids (34) and (2) the incidence of adverse events was lower, and most adverse events were reported in only one trial, thus a broad 95% CI was always obtained.

This study had several limitations. First, the severity of hearing loss are differing among included trials, while stratified data according to severity of hearing loss were not available. Second, the background therapies are varies across the included trials, which could affect the treatment effectiveness of antivirals. Third, the main cause of hearing loss are differing between adult and pediatric patients (7), whereas stratified analyses were not performed owing to smaller number of included trials. Fourth, the cause of sudden sensorineural hearing loss across included studies were not available, and the treatment effectiveness of antivirals could affect; Fifth, the treatment duration, dose of antivirals (26, 29, 30), and viral load differed among the included trials, and further stratified analyses should be performed. Finally, inherent limitations of meta-analysis based on published articles, including inevitable publication bias and restricted detailed analysis.

## Conclusions

We found that the use of antivirals significantly increased the incidence of hearing recovery in patients with hearing loss, and ganciclovir had relatively better effects on hearing recovery. Moreover, no significant differences were observed between any two types of antivirals, and the use of antivirals was well tolerated and did not increase the risk of adverse events compared with the use of a placebo. A large-scale RCT in the future should be conducted to directly compare the therapeutic effects of antivirals in the treatment of hearing loss.

## Data availability statement

The original contributions presented in the study are included in the article/supplementary material, further inquiries can be directed to the corresponding author.

## Author contributions

Conception and design and administrative support: L-LX. Provision of study materials or patients, collection and assembly of data, data analysis and interpretation, and manuscript writing: L-ML. Final approval of manuscript: L-ML and L-LX. All authors contributed to the article and approved the submitted version.
